# A Laboratory Medicine Perspective on the Investigation of Phaeochromocytoma and Paraganglioma

**DOI:** 10.3390/diagnostics13182940

**Published:** 2023-09-13

**Authors:** Christopher S. Boot

**Affiliations:** Department of Blood Sciences, Newcastle upon Tyne Hospitals NHS Foundation Trust, Newcastle upon Tyne NE1 4LP, UK; christopherboot@nhs.net

**Keywords:** phaeochromocytoma, paraganglioma, metanephrines

## Abstract

Phaeochromocytomas (PC) and sympathetic paragangliomas (PGL) are potentially malignant tumours arising from the adrenal medulla (PC) or elsewhere in the sympathetic nervous system (PGL). These tumours usually secrete catecholamines and are associated with significant morbidity and mortality, so accurate and timely diagnosis is essential. The initial diagnosis of phaeochromocytoma/paraganglioma (PPGL) is often dependent on biochemical testing. There is a range of pre-analytical, analytical and post-analytical factors influencing the analytical and diagnostic performance of biochemical tests for PPGL. Pre-analytical factors include patient preparation, sample handling and choice of test. Analytical factors include choice of methodology and the potential for analytical interference from medications and other compounds. Important factors in the post-analytical phase include provision of appropriate reference ranges, an understanding of the potential effects of various medications on metanephrine concentrations in urine and plasma and a consideration of PPGL prevalence in the patient population being tested. This article reviews these pre-analytical, analytical and post-analytical factors that must be understood in order to provide effective laboratory services for biochemical testing in the diagnosis of PPGL.

## 1. Introduction

According to the WHO classification of neuroendocrine tumours, adrenal medulla and extra-adrenal paraganglia tumours comprise phaeochromocytoma (PC), extra-adrenal paraganglioma (PGL) and neuroblastic tumours (neuroblastoma, ganglioneuroblastoma and ganglioneuroma) [1]. PC arise from chromaffin cells of the adrenal medulla, while PGL can arise from the sympathetic or parasympathetic nervous system. Sympathetic PGLs are distributed across the abdomen, usually below the diaphragm and parasympathetic PGLs are found in the head, neck or upper abdomen. All PCs and PGLs are considered to have malignant potential, although a minority of these tumours show evidence of metastasis at diagnosis [2,3,4]. PCs and sympathetic PGLs usually secrete excessive amounts of catecholamines due to overexpression of the synthetic enzymes responsible for catecholamine synthesis, while parasympathetic PGLs do not secrete catecholamines.

### 1.1. Biochemical and Genetic Characteristics of PPGL

**Figure 1:**
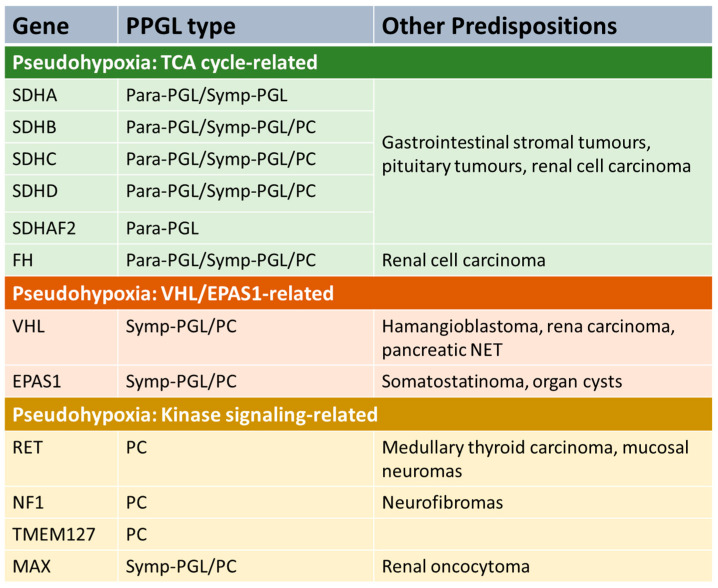
PPGL genetic pre-disposition syndromes. Para-PGL = parasympathetic PGL. Symp-PGL = sympathetic PGL. PC = phaeochromocytoma. NET = neuroendocrine tumour. TCA = tricarboxylic acid.

Phaeochromocytoma and extra-adrenal paraganglioma (PPGL) may occur sporadically, but a significant proportion (perhaps 40% [5]) is associated with an inherited predisposition syndrome. The genetics is complex, with at least 12 different genetic syndromes [5]. Genetic PPGL syndromes can be divided into several groups (Figure 1).

A number of predisposition syndromes are associated with germline mutations in genes involved in tricarboxylic acid (TCA), such as the succinate dehydrogenase subunits *SDHA*, *SDHB*, *SDHC* and *SDHD*, an assembly factor of the succinate dehydrogenase complex (*SDHAF2*) and fumarase (*FH*). Mutations in these genes lead to accumulation of metabolites such as succinate or fumarate that have oncogenic effects. Another group of PPGL syndromes are those that are linked with activation of kinase signalling pathways. The most common is gain-of-function mutations in *RET* which lead to the disorder multiple endocrine neoplasia 2 (MEN2), which is associated with increased risk of PC and other endocrinopathies (depending on sub-type of the disease), including medullary thyroid cancer and primary hyperparathyroidism [6]. Mutations in *NF1* (causing neurofibromatosis type 1), *TMEM127* and *MAX* also predispose to PPGL due to alterations in kinase signalling [7]. Another PPGL syndrome is caused by mutations in the tumour-suppressor gene *VHL* leading to Von Hippel–Lindau syndrome, which predisposes to a variety of malignant and benign tumours in various organs, including PC [8]. Mutations in *EPAS1* can also predispose to PPGL [9].

**Figure 2:**
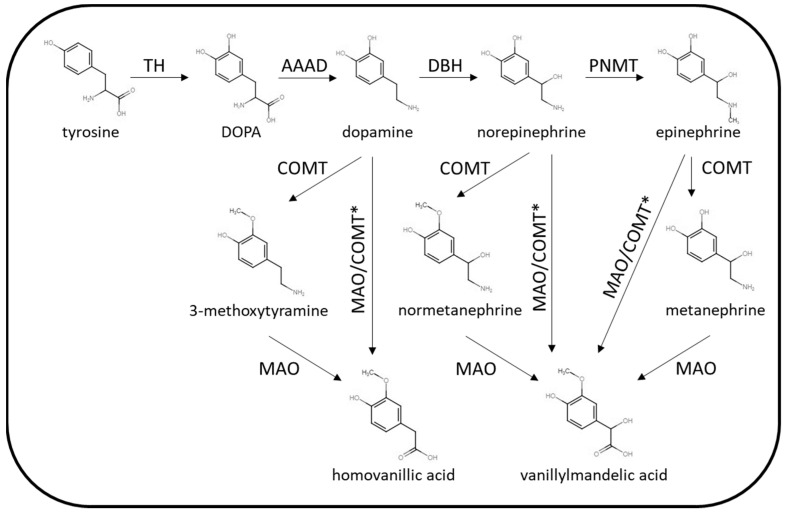
Summary of catecholamine synthetic and metabolic pathways. TH = tyrosine hydroxylase, AAAD = aromatic L-amino acid decarboxylase, DBH = dopamine β-hydroxylase, PNMT = phenylethanolamine N-methyltransferase, COMT = catechol O-methyltransferase, MAO = monoamine oxidase. * Via other intermediates.

The biochemical phenotype of PPGL is variable with the pattern of catecholamine excess dependent on location, extent of paraganglial cell differentiation and tumoral enzyme expression. The enzymes involved in catecholamine synthesis and metabolism are summarised in Figure 2.

The key synthetic enzymes that may be expressed by PPGL are tyrosine hydroxylase (TH), dopamine β-hydroxylase (DBH) and phenylethanolamine N-methyltransferase (PNMT). Parasympathetic PGLs do not express the enzymes involved in catecholamine synthesis (acetylcholine is the neurotransmitter present in the parasympathetic nervous system), and so these tumours are silent in terms of catecholamine excess. Rarely, sympathetic PPGL may also lack the enzymes required for catecholamine synthesis. Some sympathetic PPGLs are associated with excessive dopamine production without norepinephrine/epinephrine as TH is over expressed but not DBH or PNMT. This biochemical phenotype is often associated with mutations in SDH genes or *FH*. Adrenal or extra-adrenal PPGL producing excessive norepinephrine (with or without dopamine) are more common. PPGLs producing epinephrine are usually adrenal tumours that are well-differentiated with expression of PNMT. The biochemical phenotype of PPGL can vary between inherited predisposition syndromes. For example, PPGLs arising in patients with mutations in the SDH genes are more likely to harbour biochemically silent parasympathetic tumours or lesions producing only dopamine or norepinephrine [5]. PPGLs associated with mutations with *RET* or *NF1* mutations on the other hand, are more likely to produce epinephrine in addition to norepinephrine. The site of PPGL tumours may also vary according to the genetic cause, for example SDH mutations may be associated with tumours in the adrenal, abdomen or head and neck, while *RET*- and *NF1*-related tumours are usually located in the adrenal rather than other locations. While the classic symptoms of sympathetic PPGL are generally associated with catecholamine excess, PPGLs may also secrete neuropeptides in some cases. For example, cortisol excess caused by adrenocorticotrophic hormone (ACTH) or corticotrophin releasing hormone (CRH) secretion by phaeochromocytoma [10,11] has been described as has symptomatic vasoactive intestinal peptide (VIP) [12,13] and growth hormone-releasing hormone (GHRH) [14] secretion.

### 1.2. Presentation of PPGL—Symptoms, Morbidity and Mortality

Patients with suspected PPGL may present in various ways. Many patients harbouring sympathetic PPGL are investigated due to the presence of signs or symptoms that are associated with catecholamine excess. These symptoms are typically paroxysmal, varying in severity and frequency, and include hypertension, headache, palpitations, sweating and anxiety [15]. More rarely, patients can also present with orthostatic hypotension. Although these symptoms can occur spontaneously in sympathetic PPGL, they may also be provoked by certain triggers including physical/psychological stress, exercise, alcohol and a range of medications [16] (including glucocorticoids, antidepressants, β-blockers, opioid analgesics, monoamine oxidase inhibitors, tricyclic antidepressants and sympathomimetics). A significant proportion of PPGLs, however, are diagnosed in the absence of any of these symptoms. Head and neck parasympathetic PGLs do not present with catecholaminergic symptoms due to the lack of catecholamine secretion. The most significant symptoms associated with these tumours are often due to the location and mass effects of the tumour with compression and infiltration potentially leading to nerve palsies, hearing problems and dysphagia [17]. Sympathetic PPGLs may also be diagnosed in some patients without significant symptoms. Routine testing for PPGL is performed in patients known to carry pathogenic mutations in genes associated with PPGL predisposition syndromes. PPGL may be detected at an early stage in these patients, when the tumours are relatively small and so less likely to be secreting large amounts of catecholamines. The same can be said of patients who are under surveillance following treatment for a previous PPGL due to the risk of recurrence. Another group where asymptomatic PPGL may be detected is patients who have an incidentally discovered adrenal lesion or incidentaloma. Adrenal incidentalomas discovered during abdominal imaging are common, with the reported prevalence of an adrenal mass between 1 and 8% [18] (increasing with age) and incidentalomas appearing on 0.3 to 5.1% of abdominal CT scans [18] (the wide variation in reported prevalence is likely related to different settings, patient selection and exclusion criteria). According to published survey data, between 1 and 8.5% of adrenal incidentalomas prove to be phaeochromocytomas [19,20,21]. Although on detailed history-taking, many patients with incidentally discovered phaeochromocytoma have signs or symptoms associated with PPGL, some do not, and so the absence of symptoms or hypertension does not exclude phaeochromocytoma in this group of patients. A recent multi-centre series of 245 patients with PPGL found that the background for the diagnosis was presentation with signs/symptoms in 37% of cases, incidentaloma in 36% and surveillance (known susceptibility syndrome or previous PPGL) in 27% [22].

The overall prevalence of PPGL is less than 0.05% in the general population and between 0.2 to 0.6% in hypertensive patients [23]. Although these tumours are rare, it is important to exclude a diagnosis of PPGL in the patient groups described above due to the significant morbidity and mortality associated with PPGL. Cardiovascular morbidity and mortality are significant, with the risk of cardiovascular complications (myocardial infarction, stroke, transient ischaemic attacks) perhaps 10 times higher in patients with phaeochromocytoma compared to matched controls [24]. Peri-operative complications can lead to morbidity and mortality due to surgical haemodynamic instability without appropriate pre-operative preparation [25]. While this can be managed through pre-operative medical therapy, surgical procedures performed on patients with an undetected sympathetic PPGL remain high risk. In the case of non-metastatic PPGL, surgery is often curative and there is a 95% 5-year survival with recurrence in less than 5% of cases [26]. However, around 10–15% of phaeochromocytomas and up 50% of abdominal PGLs are associated with metastasis [27]. Metastatic PPGL has a lower 5-year survival rate of around 30 to 40% [28]. European guidelines recommend surveillance for at least 10 years in all patients undergoing surgery to remove a PPGL to detect recurrence and life-long follow up for those considered to be at higher risk of recurrence [29] (those with genetic susceptibility, PGL or a large tumour at diagnosis).

## 2. The Role of Biochemical Testing in PPGL

### 2.1. Diagnosis of PPGL

The diagnosis of PPGL requires a multi-disciplinary approach involving biochemistry, radiology and genetic testing. The initial investigation for patients with symptoms or a history leading to suspicion that the patient could have a PPGL usually consists of biochemical testing. Various biochemical tests have been used in the initial investigation of PPGL including measurement of catecholamines in plasma or urine and measurements of catecholamine metabolites, such as the methylated catecholamine metabolites (metanephrines) or downstream metabolites, such as vanillylmandelic acid (VMA). The biochemical tests that are available for the initial testing of PPGL have varying diagnostic characteristics and can be measured using a variety of methodologies, which will be discussed in more detail later in the article. None of the available biochemical tests provide 100% diagnostic accuracy and can produce false positive (and potentially false negative) results. Any positive biochemical test results should receive appropriate follow-up, which will depend on the extent of the elevation in the biochemical markers of PPGL, the clinical presentation and the use of medications that may affect the biochemistry results [16]. If there is biochemical evidence for the presence of a PPGL, imaging studies are recommended to confirm the diagnosis and locate the tumour [16]. In some cases, performing imaging to locate PPGL is indicated without positive biochemistry, for example, possible head and neck PGL that are biochemically silent. Endocrine Society guidelines recommend CT as an initial imaging method for localising PPGLs [16] as it provides a high degree of sensitivity and a high degree of resolution. Almost all PPGLs exhibit a mean attenuation of more than 10 Hounsfield units (HU) on unenhanced CT [30,31,32,33]. CT has lower sensitivity for metastases and for head and neck PGL compared to MRI [16]. Functional imaging is recommended for the detection of patients with suspected metastatic disease. Use of modalities such as ^68^Ga-DOTA-SSA PET/CT, ^18^F-FDG, ^18^F-FDOPA PET and ^123^I-MIBG SPECT have all been recommended [16,23]. Improved understanding of the relationship between PPGL genetics and imaging features is leading to refinements in individualised approaches to the use of molecular imaging in the detection and staging of PPGL [34].

As around 40% of patients with a diagnosis of PPGL carry a germline mutation in a susceptibility gene, consideration of genetic testing in all new cases is recommended, regardless of age and family history [16,35]. Identification of a pathogenic mutation in a susceptibility gene may inform management and post-treatment surveillance of the index case. For example, mutations in certain genes (such as *SDHB*) are associated with an increased risk of metastatic progression. Patients known to be at increased risk of metastasis may be followed-up with stricter, long-term surveillance. Some genetic syndromes are associated with the development of other tumours, for example, *VHL* with renal, pancreatic and other tumours [36]. This may inform patient surveillance, with additional imaging, biochemical testing or other investigations performed depending on the tumours associated with their inherited susceptibility syndrome [35]. The identification of a pathogenic mutation in a PPGL susceptibility gene also has implications for family members. Pre-symptomatic surveillance is recommended in known carriers of mutations in susceptibility genes as there is an opportunity for the early detection of PPGL and any other tumours carriers may be at risk of developing. The timing and frequency of surveillance and the testing performed varies between syndromes, depending on the likely risk of developing PPGL or other tumours. The testing performed typically includes biochemical screening, and regular imaging may also be recommended in syndromes where penetrance is high or there is an increased risk of malignancy [35].

### 2.2. Biochemical Tests for PPGL

A number of biochemical markers have been used in the diagnosis of PPGL, including catecholamines (epinephrine, norepinephrine and dopamine) in plasma or urine, metanephrines (the methylated metabolites metanephrine, normetanephrine and 3-methoxytyramine) in plasma or urine and the catecholamine metabolite vanillylmandelic acid in urine. These tests have been widely studied and diagnostic performance of these tests clearly varies [37,38]. In addition to choice of tests, choice of analytical methodology can also have a significant influence on the diagnostic performance of biochemical tests for PPGL. For example, metanephrines have been measured using chemical methods, immunoassay, high-performance liquid chromatography with electrochemical detection (LC-ECD) and liquid chromatography–tandem mass spectrometry (LC-MS/MS). Diagnostic as well as analytical performance may vary between methods [38]. When deciding which biochemical markers and what methodology to use, we need to consider the ideal diagnostic performance characteristics for a biochemical test used in initial testing for PPGL. Of course, the ideal test would offer 100% diagnostic accuracy, but realistically, none of the available tests offer this. Diagnostic sensitivity is a priority as PPGLs are associated with a high degree of morbidity and mortality if left undetected and untreated. The consequences of false negative results are therefore significant. This means that diagnostic thresholds for PPGL are often chosen so that they offer a high sensitivity and high negative predictive value. A certain number of false positives can be accepted to maximise sensitivity, as follow up testing (further biochemical testing and/or imaging for example) can be performed to distinguish true from false positives. However, it is also important to maintain as high a degree of diagnostic specificity as possible without compromising diagnostic sensitivity. If diagnostic specificity and positive predictive value are low, investigation of the large number of false positive results generated will be a significant burden and there will be an increased danger of over-investigation and even inappropriate treatment of patients without PPGL. When characterising the diagnostic performance for biochemical tests for PPGL, it is important to consider the underlying pre-test probability of PPGL in the populations being tested in addition to the diagnostic specificity and sensitivity of the test. Although PPGLs are relatively rare, the signs and symptoms of PPGL are common. For example, there is overlap with primary hypertension and other causes of secondary hypertension and with anxiety syndromes. This means that symptomatic patients being tested for PPGL often have a low pre-test probability due to the low prevalence in these populations. On the other hand, patients under surveillance because they have a known pathogenic mutation in a PPGL susceptibility gene or a history of treated PPGL have a significantly higher pre-test probability despite potentially being asymptomatic. The pre-test probability in the population being tested has a strong influence on the negative and positive predictive value of the test. These issues are discussed further in the Post-Analytical Considerations section later in the article.

While a range of biochemistry tests have been used in the investigation of PPGL, accumulating evidence indicates that measurement of metanephrines, either in plasma or urine offer improved diagnostic performance over measurement of catecholamines or VMA [37,38,39]. This has led to the recommendation that metanephrines, rather than other biochemical markers, are measured during biochemical testing in several guidelines and position/consensus statements [16,23,29,40,41,42]. As metanephrines are widely regarded as the biochemical markers of choice for PPGL, the rest of this review will focus on the discussion of the measurement of plasma and urine metanephrines rather than other tests. The improved diagnostic performance of metanephrines compared to catecholamines is thought to be due to the mechanisms behind the release of these compounds into the circulation. Metanephrines are produced within normal adrenal chromaffin or PPGL cells following passive leakage of catecholamines from storage vesicles into the cytoplasm. The presence of the enzyme catechol-O-methyltransferase (COMT) in the cytoplasm then leads to rapid metabolism to produce metanephrines. This process causes continuous release of metanephrines into the circulation from PPGL tissue independently of episodic, active catecholamine secretion via exocytosis of catecholamine storage vesicles [43]. This continual flux of metanephrines from PPGL tumours into the circulation accounts for their increased diagnostic sensitivity relative to measurement of the catecholamines themselves. Large studies have demonstrated that both plasma and urine metanephrines offer high specificity and sensitivity with plasma metanephrines collected with the patient in a supine posture offering optimal diagnostic performance. For example, Lenders et al. demonstrated that plasma and urine metanephrines (by LC-ECD) performed significantly better than urine catecholamines, urine VMA or total metanephrines measured by a spectrophotometric method [38]. More recently, a large study by Eisenhofer et al. [44] demonstrated that plasma metanephrines (measured by LC-MS/MS) provide a high degree of diagnostic accuracy (sensitivity 97.9%, specificity 94.2%) with urine metanephrines, while not being quite as accurate and also offering high accuracy (sensitivity 92.9%, specificity 92.1%). Although plasma/urine metanephrines have been proven to perform well as biochemical tests for PPGL, this is dependent on a range of many pre-analytical, analytical and post-analytical factors.

## 3. Pre-Analytical Considerations

### 3.1. Effects of Medications

A common factor confounding the interpretation of biochemical testing for PPGL is the effect of various medications on the measurement of catecholamines and their metabolites. In some cases, this is due to analytical interference in the methods used. For example, paracetamol and sulfasalazine (or their metabolites) can interfere with HPLC-ECD methods for urine metanephrines [45,46] and 3-O-methyldopa and midodrine have the potential to interfere with LC-MS/MS methods for metanephrines [47,48]. These true analytical interferences will be discussed in more detail in the section on analytical considerations. More common than these true analytical interferences are problems with false positive results due to effects of certain medications on the secretion or metabolism of catecholamines and/or their metabolites. There is a long list of medications that have been implicated in false-positive metanephrine/catecholamine results, and these are summarised in Table 1, along with a brief description in the mechanism for elevated results.

Common causes of these pharmacological interferences include medications which inhibit the neuronal uptake of catecholamines, such as tricyclic antidepressants (TCAs) [49]. Venlafaxine, a selective serotonin and norepinephrine reuptake inhibitor (SSNRI), has been reported to cause significantly elevated plasma normetanephrine [50]. However, selective serotonin reuptake inhibitors (SSRIs) seem less likely to cause problematic elevations [49], likely due to their lower potency as norepinephrine uptake inhibitors. Phenoxybenzamine (a non-selective α-blocker, often used to prepare patients with PPGL prior to surgery) can cause elevated plasma/urine normetanephrine through antagonism of the α1-adrenoreceptor. Selective α-blockers, such as doxazosin, terazosin and prazosin, are less likely to be associated with false positive metanephrine results (although may be associated with elevated urine catecholamine results). β-blockers such as labetalol, atenolol and propranolol may be associated with higher false positive rates for plasma metanephrine and urine metanephrine/normetanephrine [49]. Although calcium channel antagonists have been reported to cause false positives in biochemical testing for PPGL, this appears to predominantly effect measurement of the catecholamines themselves, rather than metanephrines. Sympathomimetics, such as pseudoephedrine, can cause elevated metanephrines, with measurements in urine apparently more likely to be affected than in plasma [49]. Monoamine oxidase inhibitors may also cause elevations in metanephrines by inhibiting their metabolism [51], while the Parkinson’s disease medication L-Dopa causes elevations in dopamine and its metabolite 3-methoxytyramine [52,53].

While medication-related false positives are a common problem in biochemical testing for PPGL, withdrawal of some of these medications prior to sampling can be challenging, particularly given that some medications may have a significant washout period. In most cases it may be preferable to initiate testing while on potentially problematic medications and then only consider withdrawing them if abnormal test results are causing diagnostic uncertainty. It is important that clinicians are aware of the potential impact of medications to avoid over-investigation and potentially misdiagnosis of patients being investigated for PPGL. Laboratories providing services for biochemical tests for PPGL should be able to provide clinicians with appropriate advice on the interpretation of test results in the context of potentially interfering medications and further testing in cases of potential false positives.

### 3.2. Patient Preparation

The impact of dietary factors on the concentrations of catecholamines and their metabolites in plasma and urine has been widely discussed and often causes concern due to the presence of catecholamine compounds in a range of foods (e.g., bananas, pineapples and other fruit, nuts, beans, tomatoes). In the case of the measurement of plasma free norepinephrine, epinephrine, normetanephrine and metanephrine, the impact of dietary catecholamines such as dopamine and tyramine is minimal [54,55]. However, plasma dopamine and 3-methoxytyramine can be significantly elevated following ingestion of dopamine-rich foods, and 3-methoxytyramine can also be increased following ingestion of tyramine [55]. The measurement of urine total (conjugated and free) metanephrines can be much more significantly affected by dietary catecholamine intake compared to measurements of plasma free metanephrines (or indeed urine free metanephrines) [54,55]. This is important to note as although there is some evidence that urine free metanephrines may have some advantages over total metanephrines [44], currently most routine urine metanephrine methods measure both free and conjugated fractions. The larger impact of dietary catecholamines on urine conjugated metanephrines is thought to be due to gastrointestinal SULT1A3 (a sulphotransferase enzyme) activity. In view of this impact on urine total (conjugated and free) metanephrine measurements, avoidance of catecholamine-rich foods, including a range of fruit and vegetables, is usually recommended during sample collection. If dietary restriction proves difficult and a potential source of false positive results, then measurement of plasma metanephrines after an overnight fast (to avoid dietary effects on plasma 3-methoxytyramine) may be preferred over urine measurements.

Activation of the sympathetic nervous system is another factor that can lead to elevated catecholamines and metabolites in individuals without PPGL. Any acute illness can potentially lead to increased catecholamine secretion, so elevated catecholamine/metanephrine results recorded during an episode of acute illness may require confirmation when the patient is well. A range of factors influencing sympathetic activation can also have an impact on measurements in relatively well patients. For example, plasma metanephrines are significantly lower (particularly normetanephrine) when samples are collected in a supine position relative to collection in a seated position [56]. The implications for this when considering how to collect samples for measurement of plasma or urine metanephrines are discussed below. Physical exercise has been shown to increase plasma metanephrine concentrations [57,58], so intense physical activity should be avoided immediately prior to taking blood samples and during urine collections. Psychological stress can also have an impact and patients that find the process of phlebotomy difficult may be more likely to suffer false positive results. Another factor that can cause elevated plasma metanephrine results is renal insufficiency. Measurement of plasma free metanephrines is not as severely affected as measurement of total (free and deconjugated) metanephrines, but studies indicate that the 97.5th percentiles for normetanephrine and metanephrine are around 50% higher for patients with stage 4 CKD and those requiring haemodialysis [59]. A recently published protocol suggests collecting samples from haemodialysis patients towards the end of dialysis treatment rather than prior to dialysis, as this results in significantly lower plasma metanephrines results and hence lower potential for false positive results [60].

### 3.3. Sample Collection—Urine Metanephrines

The pre-analytical considerations for urine and plasma metanephrines are summarized in Table 2. Urine metanephrines are usually measured on 24 h urine collections. As discussed above, it is important to avoid foods that may lead to elevated excretion of the metanephrines during the collection period. It is also important that clinicians are aware of the medications that may affect urine metanephrine concentrations (either through pharmacological action or analytical interference) so that they can be avoided where possible. The reliability of any test based on a 24 h urine collection is dependent on correct timing of the start and end of the collection and requires the patient to ensure that they collect all urine during this period. Twenty-four-hour urine collections are often performed poorly, with studies of urine collection accuracy estimating that up to 50% of urine collections may be inaccurate [61,62]. Issuing clinicians and patients with clear instructions on obtaining an accurate 24 h collection may improve the quality of urine collections. Another potential problem with urine collections is that catecholamine and metanephrine output may be dependent on patient activity during the collection period. As described above, factors such as physical exercise, psychological stress or illness may lead to elevated catecholamine (and therefore metanephrine) output. There is also a natural circadian variation in catecholamine excretion with lower amounts of urine catecholamines produced during the night when patients are recumbent, compared to the day [63]. A possible solution to this is the use of overnight urine collections in place of 24 h collections. Peaston et al. [64] investigated the use of an overnight collection for the measurement of urine catecholamines/metanephrines and found that measurements from overnight collections offered improved diagnostic performance relative to full 24 h collections. A more recent, larger study found that measurement of urine free metanephrines in overnight collections provided improved specificity compared to 24 h collections [65]. In addition to potential improvements in diagnostic performance, overnight collections are more convenient for the patient than 24 h collections.

Historically, the collection of urine samples for the measurement of the catecholamines and their metabolites has required the use of an acid preservative (usually hydrochloric or sulphuric acid). While the catecholamines themselves can certainly undergo degradation in non-acidified samples, there is evidence that the metanephrines are relatively stable and may not require addition of strong acids to stabilise them [66,67,68]. Current practice appears to vary in this respect. An audit of laboratory practice in the UK showed that 67% of laboratories require acidified urine collections with the remainder accepting non-acidified collections [69].

### 3.4. Sample Collection—Plasma Metanephrines

As discussed above, the impact of dietary catecholamines on measurements of plasma free metanephrines is minor compared to urine total metanephrines. Plasma 3-methoxytyramine may be increased due to consumption of dopamine-containing foods, but if this is of concern, it can be avoided by collecting samples after an overnight fast. As is the case for urine metanephrines, pharmacological interference can be an issue and problematic medications should be avoided if possible. Patient posture is an important factor affecting plasma metanephrine results, particularly normetanephrine. Plasma normetanephrine is significantly lower in samples taken after a period in a supine position compared to samples taken in a seated position [56]. This is due to decreased background activation of the sympathetic nervous system in supine individuals. There is evidence that false-positive normetanephrine results are less likely in supine samples compared to seated samples, so a higher diagnostic specificity may be achieved through improved patient preparation [70]. Diagnostic sensitivity also appears to be improved when supine sampling for plasma metanephrines is used [56,71]. In supine patients, non-specific background production of plasma metanephrines is lower, which makes it easier to detect subtle elevations due to the presence of a PPGL. Achieving this improved diagnostic sensitivity is dependent on using appropriate reference ranges based on a supine reference population [56]. The optimum time for supine rest before taking the sample is at least 20 min [16,56]. There has been some debate over whether supine sampling is necessary in all patients under investigation for PPGL [72,73], as although this is ideal, some diagnostic centres may find it difficult to provide the resources necessary for supine sampling in all patients. Some researchers have published data suggesting that seated sampling offers a high degree of diagnostic sensitivity and so have argued that seated sampling is acceptable [74,75]. A large study of the diagnostic performance of plasma and urine metanephrines in different patient groups by Eisenhofer at al [44] found that false negatives were more likely in patients undergoing surveillance due to an increased risk of PPGL (due to a genetic predisposition or history of treated PPGL) as minor elevations in plasma and urine metanephrines were more common (these patients are more likely to be harbouring small, pre-symptomatic PPGL). Patients with PPGL who were investigated due to overt symptoms or signs (e.g., hypertension, diaphoresis), on the other hand, have higher plasma metanephrines on average and are unlikely to exhibit only subtle elevations. False negatives in patients investigated due to symptoms/signs of PPGL appear to be unlikely, whether using plasma metanephrines with lower, supine-based ranges or higher seated ranges (although more false positives will be encountered with seated sampling). However, it appears that for patients being tested for surveillance purposes, supine sampling should be prioritised to ensure optimal diagnostic sensitivity.

Although the metanephrines are more stable than catecholamines, it is still important to observe appropriate sample handling conditions if pre-analytical degradation of plasma metanephrines is to be avoided. Metanephrines are usually measured in EDTA or heparinised plasma. Metanephrines are not stable in whole blood at room temperature due to the ongoing action of COMT in blood cells (converting the catecholamines to their methylated metabolites) but also due to degradation. It is possible to observe either falsely elevated or falsely low results due to this mixture of effects on stability [76,77]. Blood samples for plasma metanephrines should therefore be separated as soon as possible after collection and ideally kept on ice before and during centrifugation. Post-centrifugation, plasma samples are relatively stable at 4–8 °C (perhaps 1 to 2 days) but should be stored and transported between sites frozen if not analysed on the day of collection.

## 4. Analytical Considerations

**Figure 3:**
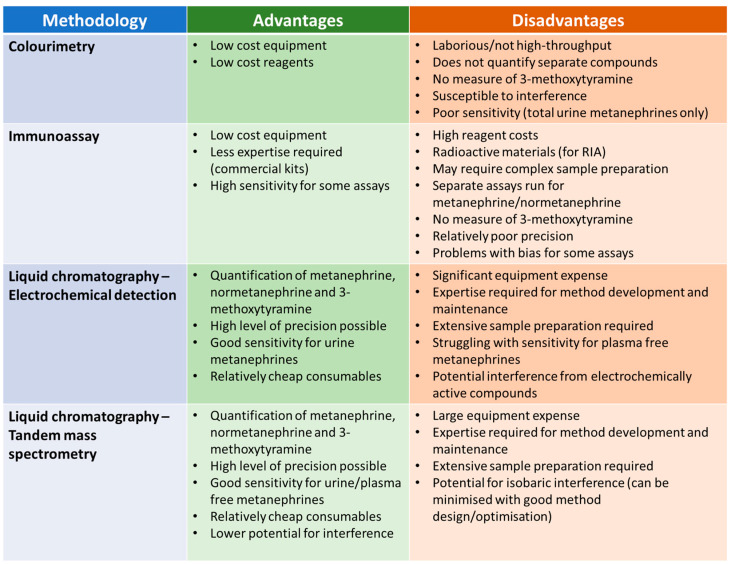
Summary of the relative advantages and disadvantages of different methodologies from measuring urine/plasma metanephrines.

Given the widespread consensus that measurement of metanephrines is preferable to catecholamines, this section will focus on the analysis of urine and plasma metanephrines. The measurement of metanephrines has evolved over the decades, with a range of methodologies applied. Each of these methodologies have relative advantages and disadvantages in terms of practicalities and analytical performance (Figure 3).

### 4.1. Colourimetry

The first methods developed for the measurement of metanephrines were colourimetric assays [78,79], which were applied to urine samples. These assays involved several steps, beginning with acid hydrolysis to liberate the conjugated fraction. A sample clean-up step was then usually required (e.g., Amberlite resin column chromatography [79]) prior to treatment with periodate to convert both metanephrine and normetanephrine to vanillin. Vanillin can be detected by spectrophotometry with an absorbance maximum at 360 nm. The principal advantages of this method are that it does not require expensive, specialist equipment and the reagents are relatively low cost. The disadvantages, however, are numerous, including a manually laborious procedure that does not lend itself to high throughput analysis, a lack of separate results for metanephrine and normetanephrine, no measurement of 3-methoxytyramine, poor analytical specificity and inadequate sensitivity for the measurement of plasma metanephrines. These methods are largely considered historic but may still be in use in some laboratories.

### 4.2. Immunoassay

Radiommunoassays (RIAs) for metanephrine and normetanephrine were developed in the 1980s [80]. These assays have been applied to the analysis of both urine and plasma, and commercial RIA kits are available [81]. Later, enzyme immunoassays for normetanephrine and metanephrine were developed which, again, are commercially available. Advantages of these assays over colourimetry include quantification of normetanephrine and metanephrine individually, rather than an integrated value, and significantly improved sensitivity. These immunoassays do not require the purchase of expensive equipment (such as LC-ECD or LC-MS/MS instruments) but are not necessarily straightforward to perform. Some of the assays include an extraction step such as column chromatography prior to analysis and some require a derivatisation step (such as acylation) to enhance the immunogenicity of the analytes. Other disadvantages include the lack of an assay for 3-methoxytyramine and poor agreement with LC-MS/MS assays, at least in some cases, which may have implications for the diagnostic performance of these immunoassays. One study investigating the performance of commercial RIA assays for urine metanephrine and normetanephrine found that there was a significant positive average bias for the metanephrine RIA relative to LC-ECD and bias for individual samples relative to LC-ECD varied widely for both metanephrine and normetanephrine RIA, with differences of up to 80% observed for both assays [82]. Both RIA assays also suffered from relatively high imprecision with interassay CVs of up to 22% for metanephrine and 16% for normetanephrine. A study of commercial ELISA assays for plasma metanephrine and normetanephrine also found significant difference in performance compared to a chromatographical method (this time LC-MS/MS) [83]. The normetanephrine and metanephrine ELISA assays gave results that were on average 60% and 39% lower, respectively, than the LC-MS/MS assay, and interassay CVs were again relatively high (11.7 to 13.0% for normetanephrine and 12.0 to 21.1% for metanephrine). The diagnostic sensitivity of the ELISA assays appeared to be significantly lower than LC-MS/MS using the manufacturer’s suggested reference ranges, but this improved when optimum diagnostic cut-off suggested by the authors of the study were used.

### 4.3. Liquid Chromatography—Electrochemical Detection

Liquid chromatography–electrochemical detection (LC-ECD) methods for urine metanephrines began to appear in the late 1970s and early 1980s. [84,85,86] These methods were a significant improvement on colourimetric methods as they provided more sensitive and specific detection of each of the individual metanephrine compounds. These methods are still widely used today for the measurement of urine metanephrines. Most LC-ECD methods have been used to measure total (i.e., unconjugated and free) urine metanephrines and so an acid hydrolysis step is required, as for colourimetric methods. It is important that this hydrolysis step is properly optimised to achieve as close to 100% recovery of the sulphated metanephrines as possible. The calibration and IQC materials used in these assays typically contain added free metanephrines, and so the hydrolysis step may not be controlled by some IQC procedures (as incomplete hydrolysis of conjugated metanephrines will have an impact on patient samples but will not affect IQC material that only contains free metanephrines) [87]. Measurement of urine free metanephrines is now gaining popularity, and there is some evidence that urine free metanephrines may offer improved diagnostic specificity compared to urine total metanephrines [44]. Although there are published methods for the measurement of free plasma metanephrines using LC-ECD [88,89], LC-MS/MS has been more widely applied to plasma samples due to the higher analytical sensitivity of the technique. Despite LC-ECD offering analytical improvements compared to colorimetric and immunoassay methods, there are some shortcomings. A barrier to the introduction of LC-ECD methods in many laboratories is the investment in analytical equipment and the expertise that is required to develop and maintain lab-developed LC based assays. In addition to the complex instrumental aspects of these methods, sample preparation is an important aspect of chromatography-based methods. Electrochemical detectors are relatively non-specific and may detect a range of compounds, leading to potential problems with interference if the chromatography step does not effectively resolve the metanephrines from potential interferents. There are many published examples of analytical interference in LC-ECD methods. Paracetamol and its metabolites have the potential to interfere in LC-ECD [46], as does sulfasalazine [45,90] (and the metabolite mesalazine), labetalol [91], amoxicillin [92] and other medications [93,94]. An unusual interference has been reported involving dietary curry leaves, which appears to cause positive interference in a commonly used internal standard (3-methoxy-4-hydroxybenzylamine) leading to falsely low metanephrine results [95]. Steps that can be taken to address interference in LC-ECD methods include optimisation of chromatography, appropriate advice on potential interferences for clinicians and patients, careful inspection of chromatograms for unusual peaks, monitoring of internal standard recovery and, ultimately, considering a more specific detector (i.e., mass spectrometry).

### 4.4. Liquid Chromatography–Tandem Mass Spectrometry

Liquid chromatography–tandem mass spectrometry (LC-MS/MS) is the most widely used method for measuring plasma metanephrines and is also becoming more widely used in the measurement of urine metanephrines (in place of LC-ECD). Detection of target compounds in LC-MS/MS is based on specific mass transitions that occur within the tandem mass spectrometer when compounds from the sample are subjected to ionisation and fragmentation. This methodology can be used to achieve low background noise and has inherently high analytical specificity (which can be further enhanced using secondary ‘qualifier’ mass transitions to confirm the identity of an analyte). The higher sensitivity that can be achieved with LC-MS/MS makes it more suitable for measurement of plasma metanephrines than other techniques. Sample preparation remains an important part of methods based on LC-MS/MS and helps to improve sensitivity, remove potentially interfering substances and reduce problems with ion suppression. The improved specificity of LC-MS/MS means that shorter run times, and hence higher throughput, can be achieved due to the appearance of fewer potential interferents and simpler chromatograms. However, although the use of LC-MS/MS addresses many of the interference problems experienced with LC-ECD methods described above, LC-MS/MS cannot be considered completely free of interference. There is potential for isobaric interference, which is where other compounds share the mass transitions that are used to detect the analytes. Such interference has been described for the medication midodrine (and desglymidodrine) [48], 3-O-methyldopa [47] and 4-hydroxy-3-methamphetamine [96]. These interferences can usually be avoided by taking them into account during method development (particularly during optimisation of chromatography) and by implementing careful inspection of chromatograms and qualifier mass transitions in routine procedures. Barriers to the uptake of LC-MS/MS in many routine laboratories include larger instrument costs than for LC-ECD and, arguably, a requirement for higher levels of expertise for method development and maintenance. Sample preparation steps are complex and time consuming, and laboratories are increasingly applying automated sample preparation, although this requires additional investment in equipment. Another important consideration is that there are potential problems with traceability and consistency between methods developed in different laboratories. There are no officially recognised international reference materials for normetanephrine, metanephrine and 3-methoxytyramine, although high purity commercial preparations are available. A range of commercial calibrators for urine and plasma metanephrines are available, which are usually prepared in a matrix designed to match patient samples as closely as possible. Differences in calibration, in addition to the large number of other variables that affect LC-MS/MS performance, have the potential to lead to sub-optimal standardisation between laboratories measuring urine and plasma metanephrines. A study evaluating the harmonisation of plasma metanephrine measurement between 12 laboratories using LC-MS/MS found that the mean bias for patient samples between methods varied form −11.6% to 16% for metanephrine and −18 to 9.5% for normetanephrine but was significantly higher for 3-methoxytyramine at −32.2% to 64% [97]. The differences in bias between participating laboratories were more significant for patient plasma samples than for external quality assessment (EQA) materials, indicating that between-lab variation might be underestimated using EQA materials that are not necessarily wholly representative of patient samples. Despite these challenges, LC-MS/MS is increasingly recognised as the method of choice for measuring metanephrines and published guidelines recommended that metanephrines are measured using LC-MS/MS or other chromatography-based methods rather than other techniques [16,23].

## 5. Post-Analytical Considerations

### 5.1. Reference Ranges

Provision of appropriate reference ranges for urine and plasma metanephrines is an essential component of a clinical laboratory service for these tests. Choice of reference ranges has a significant impact on the diagnostic performance of urine/plasma metanephrines in the diagnosis of PPGL. Use of an inappropriately high upper reference range limit (URL) may result in impaired diagnostic sensitivity and an increased false-negative rate, while use of an inappropriately low URL may result in impaired diagnostic specificity and an increased false-positive rate. Factors that may need to be taken into consideration include sex and age-related variations in urine/plasma metanephrines in healthy individuals, patient preparation (particularly patient posture for plasma metanephrines) and analytical differences between methods. Urine metanephrine output appears to vary according to sex, with higher average urinary excretion rates for normetanephrine, metanephrine and 3-methoxytyramine observed for adult males relative to females [98,99]. Urine metanephrine output also varies with age and it may be particularly important to establish age-related paediatric reference ranges as metanephrine output is higher in younger individuals particularly infants and children under 2–3 years old [100]. A survey of UK laboratories found that there was significant variation in reference ranges used for urine metanephrines [69], with the URL for normetanephrine varying from 2.88 to 5.3 μmol/24 h, metanephrine from 1.2 to 2.0 μmol/24 h and 3-methoxytyramine from 1.3 to 2.8 μmol/24 h. Some laboratories used sex- and/or age-dependent ranges, but most did not. The most likely explanation for this variation appears to be the provenance of the reference ranges with sources including in-house data ranges from published LC or LC-MS/MS kit inserts or published data. Plasma metanephrine concentrations are also subject to age relate differences, but gender appears to have a limited impact [101]. Applying age-related plasma normetanephrine reference ranges in adults may improve diagnostic performance, particularly diagnostic specificity. Plasma normetanephrine increases with age, so using a higher URL for older patients reduces the potential for false-positive results [98,101]. Posture during sample collection for the reference population also has a significant effect on plasma metanephrine reference ranges [56], with seated healthy individuals having significantly higher normetanephrine than supine individuals. The application of higher, seated reference ranges or lower supine ranges may have a significant effect on diagnostic sensitivity and specificity, although this will also depend on the posture used for collection of samples in clinical practice [56,74,75]. For example, applying lower supine ranges while collecting samples in a seated position will likely lead to a high false-positive rate, while applying higher seated ranges while collecting samples in a supine posture may lead to impaired diagnostic sensitivity [56]. Differences in applied reference ranges between laboratories could be a significant cause of variability in diagnostic performance between centres. The plasma metanephrine harmonization study by Peitzsch et al. [97] found that the use of different local reference ranges was a bigger contributor to between laboratory variability in test interpretation than differences in analytical bias between the assays employed in the 12 participating centres.

### 5.2. Interpretation

**Figure 4:**
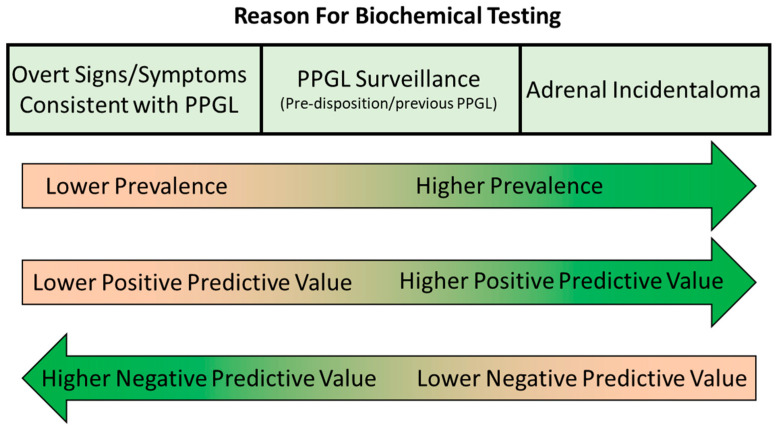
Varying prevalence, positive predictive value and negative value in biochemical testing for PPGL in different patient populations.

The final step of biochemical testing for PPGL is an appropriate interpretation of the results. When interpreting urine/plasma metanephrine results, it is important to be aware of many of the pre-analytical and analytical aspects discussed above, including potential analytical or pharmacological interference from various medications, the potential impact of dietary catecholamines and the effects of renal failure and acute illness. Clinicians requesting these investigations should have an awareness of these issues, as should laboratories providing a service for biochemical tests for PPGL so that appropriate pre- and post-testing advice can be provided if required. Key to the accurate interpretation of metanephrine results is consideration of the results in the context of other investigations and the patient’s presentation, signs/symptoms and medical history. Interpretation may vary significantly depending on the patient population being tested as the pre-test probability of the presence of a PPGL varies greatly depending on the patient’s background (Figure 4).

In cases where the patient is being tested because the patient has signs and/or symptoms that could be consistent with a PPGL, such as hypertension, sweating, anxiety, headache, etc., the pre-test probability is relatively low. These signs and symptoms are non-specific and can be present due to far more common conditions such as primary hypertension, renovascular hypertension, anxiety disorders and migraine. The low pre-test probability and higher average metanephrine concentrations in this patient group mean that diagnostic sensitivity and negative predictive value are relatively high, while specificity and positive predictive value can be limited. For example, the study of Eisenhofer et al. [44] (which included 2056 subjects, 236 with confirmed PPGL) determined a negative predictive value of 100% for plasma metanephrines and urine free metanephrines (99.9% for deconjugated metanephrines) in subjects investigated due to the presence of signs or symptoms, using the upper limit of the reference range as a diagnostic cut-off. The positive predictive value was much lower at only 58% for plasma and urine free metanephrines (47.7% for deconjugated urine metanephrines). It is important to consider the extent of the elevation of the metanephrines when interpreting these results, however. For example, in the case of patients with signs/symptoms in the Eisenhofer et al. study, using a diagnostic cut-off of double the upper limit of the reference range drastically improved the positive predictive value (from 58.1% to 97.7% for plasma metanephrines), with only a small reduction in negative predictive value (from 100.0% to 99.9% for plasma metanephrines). Therefore, the vast majority of elevated results between 1 and 2 times the upper limit of the reference range were false positives in subjects with signs/symptoms. This is because patients with PPGL are more likely to be harbouring a relatively large mass, producing significant amounts of catecholamines, and hence marked elevations in plasma/urine metanephrines, when they are at the symptomatic stage.

In cases where the patient is being tested, not necessarily because they are symptomatic, but because they either have a known pre-disposition or a known adrenal mass, the pre-test probability of PPGL is higher than for patients investigated due to the presence of signs/symptoms. Patients falling into this ‘high prevalence’ category are those under surveillance due to a known genetic pre-disposition syndrome, those under surveillance following treatment for PPGL and patients with an adrenal incidentaloma. This higher pre-test probability leads to a higher positive predictive value for elevated metanephrines results but a lower negative predictive value. PPGL lesions in this patient group are more likely to be smaller tumours at a pre-symptomatic stage and so associated with less marked metanephrine elevations in some cases than patients with overt signs/symptoms. In the study of Eisenhofer et al. [44] mentioned in the previous paragraph, the sensitivity and negative predictive value were slightly lower for patients in these categories compared to those with overt signs/symptoms. In the case of plasma metanephrines, using the upper limit of the reference range as a cut-off, negative predictive value was 99.1% (and sensitivity 96.7%) for the high prevalence groups compared to 100% for those with signs/symptoms. Positive predictive value was higher though at 76.7% compared to 58.1%. Consideration of the extent of the elevation in metanephrines should again be considered during interpretation, but it is important to consider that subtle elevations in metanephrines are more likely in true PPGL cases in this population with higher pre-test probability. Increasing the diagnostic cut-off to two times the upper limit of the reference range in the Eisenhofer et al. study [44] significantly impaired diagnostic sensitivity in this group, from 96.7% to 80% for plasma metanephrines and from 89.5% to 66.4% for urine deconjugated metanephrines. Therefore, ‘borderline’ results between one and two times the upper limit of the reference range are more likely to be observed in patients with PPGL in the population with high pre-test probability, and false negatives (i.e., results within the reference ranges) are also more likely. In patients being tested for PPGL due to the incidental discovery of an adrenal lesion, it should be remembered that the radiological characteristics can provide useful diagnostic information in addition to biochemical investigations. Almost all PPGLs exhibit a mean attenuation of more than 10 Hounsfield units (HU) on unenhanced CT [30,31,32,33], so attenuation of less than 10 HU provides a useful cut-off for ruling out PPGL [102].

While accounting for the patient background and the associated pre-test probability when considering the results of biochemical tests for PPGL can improve interpretation, there will still be a significant number of cases where there is diagnostic uncertainty. It is therefore important to have follow-up testing strategies in place. In cases where the optimal test of plasma metanephrines collected in the supine posture has not been used (e.g., seated plasma metanephrines or urine metanephrines), a follow-up test of plasma metanephrines collected under these ideal conditions may reduce the false-positive rate and allow rule-out of PPGL in patients with initial equivocal results [70]. The clonidine suppression test has also been suggested as a second-line biochemical test. Although suppression of normetanephrine upon administration of clonidine has potential as a useful test for ruling out PPGL [49,103], the test has not yet been formally recommended in evidence-based guidelines for the investigation of PPGL [16]. Chromogranin A has also been studied as a complementary test for PPGL, but the evidence supporting the utility of CgA in routine testing for PPGL is relatively poor. Most of the published studies investigating the diagnostic performance of CgA are small, and those that offer a direct comparison with metanephrines suggest that CgA has lower diagnostic sensitivity [104,105,106,107]. The specificity of CgA is also limited. CgA can be markedly elevated in patients taking proton pump inhibitors and patients with renal failure [108]. The only situation where CgA has been clearly recommended in the investigation of PPGL is as a potential biomarker for PPGLs that do not produce catecholamines. The European Society of Endocrinology guidelines recommend that CgA is measured in PPGL with negative metanephrines pre-operatively. In cases where CgA is positive, it may be monitored postoperatively to aid detection of recurrence [29]. Due to the shortcomings in the performance of biochemical tests for PPGL, it is inevitable that some cases with equivocal biochemistry will require radiological testing to determine if there is a tumour present.

## 6. Conclusions

Biochemical testing is a vital component of the diagnostic pathway for PPGL. A wide range of testing options is available in terms of analytes, sample types and analytical methodologies. Over the years, a consensus has emerged that the preferred biomarkers are the metanephrines either in urine or plasma and that chromatographical methods (particularly LC-MS/MS) perform better than other techniques. Despite improvements in the analytical performance of plasma/urine metanephrine methods, a range of pre-analytical, analytical and post-analytical factors still require careful consideration when initiating testing, performing analysis and interpreting the results. Clinicians requesting biochemical testing for PPGL and laboratories offering these tests should have a good understanding of these issues so that optimal diagnostic performance can be achieved.

## Figures and Tables

**Table 1:** Summary of potential effects of medications of measurement of metanephrines.

Pharmacolgical Effects		
Medications	Mechanism of Effects on Catecholamines/Metanephrines	Potential Impact on Results
Tricyclic antidepressants Venlafaxine	Inhibition of neuronal uptake of norepinephrine	Increased norepinephrine/normetanephrine
Phenoxybenzamine	Antagonism of α-adrenoceptors	Increased norepinephrine/normetanephrine
Selective α-adrenoceptor blockers (e.g., doxazosin)	Antagonism of α-adrenoceptors	May increase norepinephrine, but minimal effects on urine/plasma metanephrines
Monoamine oxidase inhibitors	Impaired metabolism of O-methylated catecholamine metabolites	Increased urine/plasma metanephrines
Atypical antipsychotics (e.g., quetiapine, clozapine, risperidone)	Increased secretion of norepinephrine	Increased norepinephrine/normetanephrine
Stimulants (e.g., nicotine, caffeine)	Increased secretion of epinephrine/norepinephrine	Increased epinephrine/metanephrine and/or norepinephrine/normetanephrine
Sympathomimetics (e.g., amphetamine, ephedrine)	Release of vesicular monoamines, activation of adrenoreceptors	Increased catecholamines/metanephrines
**Analytical Interference**		
**Medications**	**Methodology Potentially Affected**	Potential Impact on Results *
Paracetamol	HPLC-ECD	Increased normetanephrine/metanephrine
Sulfasalazine	HPLC-ECD	Increased normetanephrine/metanephrine
Labetalol	HPLC-ECD	Increased normetanephrine/metanephrine
Amoxicillin	HPLC-ECD	Increased normetanephrine/metanephrine
Curry leaves	HPLC-ECD (over-recovery of internal standard)	Decreased metanephrines
Midodrine (desglymidodrine)	LC-MS/MS	Increased metanephrine and 3-methoxytyramine
3-O-methydopa	LC-MS/MS	Increased 3-methoxytyramine

* Note that the impact of these analytical interferents may vary between different LC-ECD or LC-MS/MS methods.

**Table 2:** Summary pre-analytical considerations for plasma and urine metanephrines.

Pre-Analytical Considerations		
	Plasma Metanephrines	Urine Metanephrines
Diet	Dietary catecholamines have minimal effect on NMN and MN but may cause elevations in 3MT. Fasted sampling recommended.	Dietary catecholamines are more likely to affect measurement of total urine metanephrines. Exclusion of catecholamine-rich foods during sample colleciton recommended.
Medications	A range of medications can cause change in metanephrines concentrations through their pharmacological action. The potential for analytical interference is method dependent (e.g., LC-ECD vs. LC-MS/MS).	A range of medications can cause change in metanephrines concentrations through their pharmacological action. The potential for analytical interference is method dependent (e.g., LC-ECD vs. LC-MS/MS).
Physical activity/stress	Intense physical activity and physical/psychological stress can increase plasma metanephrines. This should be minimised where possible prior to phlebotomy.	Intense physical activity and physical/psychological stress can increase urine metanephrines. This should be minimised where possible throughout the period of urine collection.
Patient Posture	Supine sampling is preferred as this improves diagnostic performance compared to seated sampling.	N/A
Accuracy of sample collection	N/A	A significant proportion of 24 h urine collections may suffer from inaccurate timing.
Sample stability	Blood samples must be centrifuged ASAP following sample collection. Plasma should be stored and transported frozen if analysis does not take place on the day of collection.	Urine samples are stable for several days at room temperature. Acidification improves sample stability.

NMN = normetanephrine, MN = metanephrine, 3MT = 3-methoxytyramine, LC-ECD = liquid chromatography-electrochemical detection, LC-MS/MS = liquid chromatography–tandem mass spectrometry.

## Data Availability

Not applicable.
